# Preparation of PDA-GO/CS composite scaffold and its effects on the biological properties of human dental pulp stem cells

**DOI:** 10.1186/s12903-023-03849-4

**Published:** 2024-01-31

**Authors:** Yaoyao Li, Xinhui Huang, Weihao Fu, Zonghao Zhang, Kuancheng Xiao, Hongbing Lv

**Affiliations:** 1 Fujian Key Laboratory of Oral Diseases & Fujian Provincial Engineering Research Center of Oral Biomaterial & Stomatological Key laboratory of Fujian College and University, School and Hospital of Stomatology, Fujian Medical University, Fuzhou, Fujian People’s Republic of China; 2 School and Hospital of Stomatology, Fujian Medical University, Fuzhou, Fujian People’s Republic of China

**Keywords:** Maxillofacial bone tissue regeneration, Human dental pulp stem cells, Chitosan scaffold, Reduced graphene oxide, PDA-GO/CS composite scaffolds, Tissue engineering

## Abstract

Reduced graphene oxide (rGO) is an graphene oxide (GO) derivative of graphene, which has a large specific surface area and exhibited satisfactory physicochemical characteristics. In this experiment, GO was reduced by PDA to generate PDA-GO complex, and then PDA-GO was combined with Chitosan (CS) to synthesize PDA-GO/CS composite scaffold. PDA-GO was added to CS to improve the degradation rate of CS, and it was hoped that PDA-GO/CS composite scaffolds could be used in bone tissue engineering. Physicochemical and antimicrobial properties of the different composite scaffolds were examined to find the optimal mass fraction. Besides, we examined the scaffold’s biocompatibility by Phalloidin staining and Live and Dead fluorescent staining.

Finally, we applied ALP staining, RT-qPCR, and Alizarin red S staining to detect the effect of PDA-GO/CS on the osteogenic differentiation of human dental pulp stem cells (hDPSCs). The results showed that PDA-GO composite was successfully prepared and PDA-GO/CS composite scaffold was synthesized by combining PDA-GO with CS. Among them, 0.3%PDA-GO/CS scaffolds improves the antibacterial activity and hydrophilicity of CS, while reducing the degradation rate. In vitro, PDA-GO/CS has superior biocompatibility and enhances the early proliferation, migration and osteogenic differentiation of hDPSCs. In conclusion, PDA-GO/CS is a new scaffold materialsuitable for cell culture and has promising application prospect as scaffold for bone tissue engineering.

## Introduction

Due to the particularity of the oral and maxillofacial region, defects in facial bone tissue caused by trauma, tumors, or odontogenic infections not only affect patients physiologically but also have psychological implications. In cases of extensive and complex bone defects, clinical repair often involves autologous bone transplantation, which, however, leads to secondary trauma at the donor site. While allogeneic bone transplantation can address these issues, concerns arise regarding immune rejection and pathogen transmission, posing common challenges in the clinical reconstruction of maxillofacial bone defects. Bone tissue regeneration presents a novel approach wherein externally constructed tissues replace damaged or missing tissues to restore the form and function of deficient bone tissue in patients [1]. Recent advancements in Bone Tissue Engineering (BTE) technology are poised to effectively address current issues, offering hope to patients and establishing itself as a focal area of current research [2–4].

Seed cells are crucial components in bone tissue engineering for repairing bone defects in the oral and maxillofacial region. Currently, research focuses on Human Dental Pulp Stem Cells (hDPSCs) and Bone Marrow-Derived Mesenchymal Stem Cells (BMSCs) [5–9]. However, extracting BMSCs from bone marrow inflicts considerable damage to the body and yields a relatively limited quantity of cells [10]. Compared to BMSCs, hDPSCs possess numerous advantages. hDPSCs have a wide range of sources, obtainable from the dental pulp of impacted or orthodontic teeth extracted from patients, as well as from the pulp tissue affected by irreversible pulpitis [11, 12]. Additionally, patients’ hDPSCs can be stored long-term in liquid nitrogen, providing a readily available supply [11, 12]. hDPSCs exhibit multipotent differentiation capabilities towards odontogenesis, osteogenesis, neurogenesis and adipogenic differentiation [13–15]. The multipotent nature of hDPSCs renders the use of autologous stem cells feasible for repairing maxillofacial bone tissue.

In order for hDPSCs to exert their stemness, an appropriate extracellular matrix (ECM) is essential for their functionality. Scaffold materials serve as temporary substitutes for the ECM and are employed in bone tissue regeneration [16–18]. How to provide an excellent ECM for seed cells, enabling a more effective utilization of their potential, has become an increasingly emphasized direction among researchers in recent years. Scaffold materials represent a crucial component in bone tissue engineering, capable of inducing specific cellular functions and guiding cell proliferation and osteogenic differentiation [19]. The ideal scaffold material should exhibit high biocompatibility, appropriate porosity, adequate mechanical strength, and hydrophilicity [20]. On the other hand, for the clinical application of scaffold materials, they should also possess antibacterial properties. This characteristic is crucial in bone tissue engineering as it relates to complications associated with bone defects, particularly open fractures, where complications arising from infections exceed 30% [21], underscoring the significance of antibacterial attributes.Chitosan (CS), sourced from the chitin layers of crustaceans like crabs and shrimp, stands as a common scaffold material in tissue engineering [22]. It boasts natural polymer characteristics—Widely sourced, ease of access, non-toxicity, and biodegradability [23]. Moreover, it exhibits significant antibacterial activity against most bacteria and fungi [24]. However, its relatively rapid degradation rate [25] has restricted its use in bone tissue engineering. To address this limitation, various composite scaffolds have emerged by combining CS with other materials to enhance its properties. Examples include CS/gelatin, silk fibroin/chitosan/β-tricalcium phosphate, hydroxyapatite/CS, chitin/CS, among others, all applied in bone tissue engineering endeavors [26–29].

Graphene [30] is a unique nanomaterial composed of a layer of sp^2^ hybrid carbon atoms arranged in a honeycomb lattice. Its outstanding electrochemical properties, high surface area, and mechanical strength render it applicable across various fields, including biomedical applications [31]. Meanwhile, graphene oxide(GO) can demonstrate antibacterial mechanisms different from traditional antibiotics, granting it powerful antibacterial properties [32, 33]. Reduced Graphene Oxide (rGO) stands as one of graphene’s common derived forms, boasting a larger surface area and excellent physicochemical properties. Due to the reduction of oxygen-containing groups in rGO compared to Graphene Oxide (GO), it enhances the material’s conductivity, favoring stem cell growth. Dmitriy A. Dikin et al. [34, 35] discovered that reduced rGO exhibits increased surface area and remarkable mechanical strength post-reduction. In comparison to GO, rGO demonstrates superior biocompatibility [36, 37]. Jong Ho Lee et al. [38] found that the nanocomposite of rGO and hydroxyapatite (HAp) (rGO/HAp NCs) promotes osteogenesis in pre-osteoblastic MC3T3-E1 cells, facilitating new bone formation. Sajad Bahrami et al. [39] synthesized collagen/reduced graphene oxide (Col-rGO) composite scaffolds, where the rGO coating increased the mechanical strength of Col-rGO scaffolds by 2.8 times compared to Col scaffolds. Furthermore, the Col-rGO scaffolds demonstrated that the addition of rGO did not induce cytotoxic effects, enhancing the survival and proliferation capabilities of human BMSCs through enhanced 3D adhesion and spreading.GO can be reduced to rGO through electrochemical, thermal, or chemical methods. Among these, the most commonly used reducing agent is hydrazine, yet due to its high toxicity, an alternative, non-toxic reducing agent, polydopamine (PDA), has been chosen [40]. PDA serves as a green and non-toxic agent capable of reducing GO to rGO.

Inspired by the strong adhesion properties of mussels in nature, in 2007, researchers explored PDA due to its molecular structure resembling the adhesive interface of mussels [41]. Its key advantage lies in its ability, like mussels, to easily deposit on nearly all types of inorganic and organic substrates. PDA demonstrates excellent properties [42, 43], such as remarkable hydrophilicity, adhesiveness, non-toxicity, degradability, and biocompatibility. Studies by Liu et al. [44] revealed that a composite coating of PDA and hyaluronic acid enhanced the adhesion, growth, proliferation, and differentiation of dental pulp stem cells. Additionally, Yan Li et al. [43] observed improved hydrophilicity in polyphthalamide and polylactic acid reinforced materials with PDA coatings, promoting proliferation, adhesion, and osteogenic differentiation of mouse osteoblasts.

This study aims to synthesize PDA-GO by reacting PDA with GO, followed by its reaction with CS to fabricate PDA-GO/CS composite scaffolds. The investigation will focus on assessing the antibacterial properties and physicochemical characteristics of PDA-GO/CS composite scaffolds with varying mass fractions to determine the optimal ratio. Furthermore, it aims to explore the impact of these composite scaffolds on the proliferation, migration, and osteogenic differentiation of hDPSCs. This research endeavors to provide fundamental insights into the application of PDA-GO/CS in the field of regenerative medicine.

## Materials and methods

### Construction of PDA-GO/CS composite scaffold

1. The preparation process of PDA-GO is as follows:

Weigh a specific amount of Tris powder (Macklin Biochemical Co., Ltd)and dissolve it in triple-distilled water to prepare a 10 mM solution. Adjust the solution’s pH to 8.5 using dilute hydrochloric acid (HCl) (Aladdin Reagent Co., LTD., China).Add the corresponding amount of GO to the Tris-HCl buffer solution, stir at 500 rpm at room temperature for 20 minutes, and sonicate for 30 minutes to obtain a homogeneous GO dispersion with a concentration of 1.0 mg/mL.PDA (Aladdin Reagent Co., LTD., China) was added to the dispersing solution to ensure that the final concentration of PDA was 1.0 mg/mL. The mixture was stirred at 600 rpm at room temperature for 18 h.

As the reaction between PDA and GO progresses, the color of the mixture gradually darkens, transitioning from the initial brown color of GO to a black-colored PDA-GO. Next, the obtained PDA-GO dispersion is centrifuged and washed with anhydrous ethanol and deionized water until the supernatant turns colorless. Then, it is freeze-dried in a freeze dryer for 24 hours.The resultant complex is denoted as PDA-GO.

(2) The preparation process of PDA-GO/CS is as follows:

Add the CS (Sinopsin Chemical Reagent Co., LTD., China) to an appropriate volume of a 2.5% acetic acid solution, and stir at room temperature at 1000 rpm for 4 hours until it completely dissolves, forming a CS solution with a concentration of 20.0 mg/mL.A certain amount of CS solution (20.0 mg/mL) was added into the PD-GO dispersion solution, and the system was mixed evenly with constant stirring. The mass fraction of PD-GO was 0.1, 0.3, 0.5, 0.7% PD-GO /CS composite scaffold, respectively. After sonication for 10 minutes, heat the PDA-GO/CS mixture at 500 rpm, simultaneously stirring, to a temperature of 90 °C and maintain it for 2 hours. Place the mixed dispersion in a low-temperature freezer and freeze it at − 50 °C for 48 hours to ensure complete freezing of the system. Transfer the fully frozen solid into a vacuum freeze dryer for 48 hours to generate the PDA-GO/CS composite scaffold using the freeze-drying method. Soak the obtained PDA-GO/CS composite scaffold in a sodium hydroxide solution for 5 minutes to adjust the pH. Then, rinse it several times with alternating washes of deionized water and anhydrous ethanol to remove impurities, ensuring the pH of the composite scaffold is within a neutral range. Place the fully frozen solid of the PDA-GO/CS composite scaffold in a vacuum freeze dryer for 24 hours to generate the scaffold using the freeze-drying method.

(3) The preparation process of GO/CS is as follows:

Take a certain amount of CS solution (20.0 mg/mL) and add it into the above GO dispersion solution, and keep stirring to ensure even mixing of the system. Place the mixture in a low temperature refrigerator as described above.

### Detection of PDA-GO/CS composite scaffold

#### Fourier infrared detection of functional groups of the support

The functional groups of the scaffolds were tested by Fourier infrared spectrometer with wave number range of 500–4000 cm^−1^ and resolution of 4 cm^−1^. During sample preparation, potassium bromide (KBr) was dried in advance and ground into a uniform powder, and then a small amount of sample was added (the mass ratio of sample to KBr was controlled between 1:30–1:200) for full grinding. After evenly mixed, the tablet was pressed under 60 Mpa and the pressure was applied for about 2 min.

#### Detection of antibacterial property of scaffolds

Five groups of 0.1, 0.3, 0.5, 0.7% PD-GO /CS composite stents and CS stents were placed in a 24-well plate, and 100 μl (OD = 0.25) *Staphylococcus aureus* (S.aureus, ATCC 25293) was added to each well. Colony Forming Units (CFU) can be counted by forming single colonies after 18 h.

#### Observation and detection of scaffold microstructure

After freeze-drying, the sections of each group of scaffolds were treated with gold spray, and the influence of the internal microstructure of the five groups of scaffolds was observed by scanning electron microscopy. The average aperture size of each group of scaffolds was analyzed by Image J software based on the differences in the internal microstructure of the five groups of scaffolds under scanning electron microscope.

#### Measurement of swelling ratio of support

After freeze-drying, the five groups of supports were weighed as m0, and then fully immersed in the same amount of deionized water, respectively. After 24 hours, they were taken out, and the filter paper gently sucked up the surface moisture of the supports, and weighed again as m1. For each group of samples, make 3 parallel samples, and the Swelling Ratio of the five groups of supports shall be calculated using the following formula:

1 $$\textrm{Swelling}\ \textrm{Ratio}=\frac{\textrm{m}1-\textrm{m}0}{\textrm{m}0}\times 100\%$$

#### Scaffold porosity test

The weight of the specific gravity flask filled with anhydrous ethanol was denoted as m1, and the weight of the freeze-dried m0 support was put into the specific gravity flask, ultrasonic shock was carried out, and then the anhydrous ethanol was filled again without obvious bubbles, and the weight was denoted as m2. Take out the support soaked in anhydrous ethanol, weigh the weight of the specific gravity bottle and the remaining anhydrous ethanol, recorded as m3; Three parallel samples were made for each group, and the Porosity Ratio of each group was calculated by the following formula:

2 $$\textrm{Porosity}\ \textrm{Ratio}=\frac{\textrm{m}2-\textrm{m}3-\textrm{m}0}{\textrm{m}1-\textrm{m}3}\times 100\%$$

#### In vitro degradation detection of scaffolds

The freeze-dried scaffolds of each group were weighed as w0, and were completely soaked in PBS solution containing 0.5 g/L lysozyme, and cultured in a 37 °C incubator. The scaffolds were taken out after the 7th, 14th and 21d, respectively. After the materials were taken out, they were repeatedly rinsed with the same amount of deionized water for 3 times, then freeze-dried again, and weighed w1. There were 3 parallel samples in each group, and Weight Loss(%) of each group was calculated by the following formula:

3 $$\textrm{Weight}\ \textrm{Loss}\left(\%\right)=\frac{\textrm{w}0-\textrm{w}1}{\textrm{w}0}\times 100\%$$

### Preparation of dental pulp stem cells

Dental pulp stem cells were obtained from healthy premolars or third molars (13–23 years old) without caries and periodontitis with the permission of the Ethics Committee of the Affiliated Stomatological Hospital of Fujian Medical University and patients’informed approval. Isolated hDPSCs according to published procedures and cultivated in DMEM supplemented with 10% FBS and 1% P/S. All cells were kept in an incubator containing 5% CO_2_, 37 °C, and passaged for 3–5 generations for subsequent experiments. To identify the stemness of dental pulp cells, we inspected the cell genesis by immunofluorescence (IF) staining with antibodies Vimentin and CK-14 and through the CCK-8 method to detect the cells’ self-renewal proliferative capacity. Furthermore, according to the requirements of manufacturers, Alizarin Red S (Sigma, USA), Oil Red O (Cyagen, China) were stained to identify osteogenic and lipogenic multidirectional differentiation characteristics.

1. Immunoidentification of cells

P3 cells were selected and inoculated into 24-well plates at a density of 3 × 10^3^ cells per well and placed in cell incubators overnight. On the second day, the culture medium was removed and fixed for 15 min with 4% paraformaldehyde solution, 0.3% TritonX-100 solution for membrane breaking, and 5% BSA(500 μL/ well) solution for cell sealing. Adequate amounts of rabbit anti-human Vimentin antibody and rabbit anti-human cytokeratin (ck-14) antibody were added to the well plate according to the instructions. Nucleus staining: DAPI was added and incubated against light for 5 min for nuclear restaining, and PBS was washed 3 times, 5 min/ time. Images were then observed and collected under a fluorescent inverted microscope.

(2) Cell growth curve

P3 cells were selected and inoculated into 96-well plates at a density of 3 × 10^3^ cells/well. 7 groups were set with 6 multiple Wells in each group. 100 μL complete medium was added to each well. The culture was cultured at 37 °C, 5%CO_2_ and saturated humidity, and the liquid was changed every 3 days. At 1, 2, 3, 4, 5, 6, 7 d, CCK-8 (Dojindo, Japan) method was used to determine the results. 10 μL CCK-8 reagent + 90 μL incomplete medium was added to each well, gently mixed, and incubated in a cell incubator for 1 h away from light. OD values at 450 nm were measured on an enzyme-labeled instrument, and 7-day data were collected to plot the growth curve.

(3) Flow cytometry of cells

P3 hDPSCs in good growth state were taken, digested with 0.25% trypsin, centrifuged at 4 °C, centrifuged at 1000 rpm for 5 min, cleaned with PBS for 3 times, cell suspension was made, counted with cell counting plates, and diluted. Six 1 × 10^6^/mL cell suspensions were placed in flow cytometry tubes, of which 2 were control groups and the other 4 were CD34, CD45, CD73 and CD90 (Sigma, USA) detection groups, respectively. The 6 groups of cells were centrifuged and re-suspended with PBS for 3 times. After the last centrifugation, the supernatant was abandoned and 50 μL PBS was added, and 1 μL corresponding antibody was added to the 4 test groups, and incubated at 4 °C for 30 min, away from light. The cells were placed into the upper groove of flow cytometer in turn, and detected by flow cytometer to analyze the ratio of fluorescence positive cells.

(4) Alizarin red staining

The P3 cells were inoculated into 12-well plates with 5 × 10^4^ cells per well, the cell growth status was observed every day, and the fluid was changed once every 3 days. When the cell density in 12-well plates was observed to increase to 70%, the original complete medium was discarded and replaced with mineralized induction solution, which was routinely cultured in a cell incubator at 37 °C and 5%CO_2_. Replace the fresh mineralized induction solution every 2 days. After 21 days of induction culture, alizarin red staining was performed. The original complete medium was removed, rinsed with PBS twice, fixed with 4% paraformaldehyde for 30 min, rinsed with PBS three times, and then stained with 1% pH 4.1 alizarin red staining solution for 10 min. After rinsing with three steaming water, the formation of calcified nodules was observed under an inverted microscope and photographed.

(5) Oil red O staining

The P3 cells were inoculated into 12-well plates at 5 × 10^4^ cells per well, and the growth status of the cells was observed every day, and the fluid was changed once every 3 days. When cells were observed to be close to the bottom of the porous plates, the original complete medium was discarded and replaced with lipid-induced differentiation after 21 days of culture according to the instructions, oil red O staining (Sigma, USA) was performed. The original lipid-induced differentiation medium was removed, rinsed with PBS twice, fixed with 4% paraformaldehyde for 30 min, rinsed with PBS three times, and then stained with oil red O staining solution for 30 min. After rinsing with three steaming water, the staining was observed under an inverted microscope and photographed.

### Live and dead fluorescence staining

P3 cells were selected and inoculated into 96-well plates at a cell density of 2 × 10^3^ cells/well. 24 h later, the sample extract was changed (refer to GB/T 16886.12–2005). Two groups, the Control group and 0.3% PDA-GO/CS group, were cultured in cell incubators. Each well was washed twice with PBS, and each well was added with live and dead fluorescent stain. The well was incubated for 10 min at room temperature, and then observed and photographed under a fluorescence microscope.

### Rhodamine - ghost pen cyclic peptide staining

P3 cells were selected and inoculated into 96-well plates at a cell density of 1 × 10^3^ cells/well. 24 h later, the sample extract was changed, and the Control group and 0.3% PDA-GO/CS group were placed in cell incubators for culture. After 3 days, the cells were taken out for fluorescence staining according to the instructions. Finally, they were observed and photographed under fluorescent inverted microscope.

### Effects of PDA-GO/CS on proliferation capacity of hDPSCs

The experiment was divided into two groups: Control group and 0.3%PDA-GO/CS. The proliferation of hDPSCs was determined by CCK-8 method. The cell density of the prepared cell suspension was measured by cell count plate, and the cell density of 3 × 10^3^ cells per well was inoculated into the 96-well plate. On the 2nd day, the sample extract was replaced and continued to be cultured in the incubator, and the solution was changed every 2 days. A board was taken out for detection at the 1st, 3rd and 5th days, and OD values of each group were measured.

### Effects of PDA-GO/CS on hDPSCs migration

The group of experiments was the same as above. The cells were inoculated into 12-well plates at a density of 6 × 10^4^ cells per well and cultured in α-MEM medium with 10%FBS for 1 day. On the second day, the cells were observed under the microscope to cover the bottom of the orifice plate, which could be scratched. After the scratch, wash twice with PBS and shake gently to remove the scratched cells. Different groups of scaffold extracts were added, photographed under the microscope and recorded as 0 h. After that, the photos were taken every 6 hours, and if the cell migration was slow, the photos could be taken once in 12 hours, and the same areas were taken each time. After the cells basically covered the scratched area, the scratched area of each picture was calculated, and the migration rate was calculated according to formula (4).

4 $$\textrm{mobility}=\frac{0\textrm{h}\ \textrm{scratch}\ \textrm{area}-\textrm{Scratch}\ \textrm{area}\ \textrm{in}\ \textrm{a}\ \textrm{certain}\ \textrm{period}\ \textrm{of}\ \textrm{time}}{0\textrm{h}\ \textrm{scratch}\ \textrm{area}}\times 100\%.$$

### Effects of PDA-GO/CS on osteogenic differentiation of hDPSCs

1. Alkaline phosphatase staining

The group of experiments wasthe same as above. The extraction solution was prepared. The difference was that in this experiment, osteogenic induction solution was used to soak the scaffold, and a blank control group was also set and placed in a cell incubator. The osteogenic induction solution was collected every 2 days. Cells were inoculated into 12-well plates at 3 × 10^4^ cells per well. When cell density in 12-well plates increased to 70%, the original complete medium was discarded and replaced with mineralized induction solution. The original medium was discarded on the 7th and 14th day after induction, and alkaline phosphatase staining was performed according to the reagent manufacturer’s instructions, and the general view was taken under natural light. Look and photograph under an inverted microscope.

(2) Alizarin red staining

The group of experiments is the same as above. The cells were inoculated into 12-well plates at the cell volume of 3 × 10^4^/well. When the cell density in 12-well plates increased to 75%, the original complete medium was discarded and replaced with mineralized induction solution. After 21 days of induction culture, alizarin red staining was performed according to the reagent supplier’s instructions. The formation of calcified nodules was observed and photographed under an inverted microscope.

(3) Real-time fluorescence quantitative PCR experiment

The cell scaffold complex was formed by the above method. After 7 and 14 days of culture, the expression of genes related to osteogenic differentiation was collected and analyzed. Total RNA was extracted using Nuclezol (Invitrogen, USA) according to manufacturer’s instructions. cDNA is then reversely transcribed from total RNA using reverse transcriptase (Takara, Japan). After cDNA was obtained, the expression levels of osteogenic genes RUNX2, ALP, OSX, COL-1 and OCN were detected by RT-qPCR using GAPDH gene as control. Table 1 shows the primers used to amplify all the genes.

**Table 1:** Primer sequences used in real-time PCR assay

Genes	Primer sequences
GAPDH	5′- CAGGAGGCATTGCTGATGAT − 3’
	5′- GAAGGCTGGGGCTCATTT − 3’
RUNX2	5′- CACAAGTGCGGTGCAAACTT − 3’
	5′- TGCTTGCAGCCTTAAATGACT − 3”
OSX	5′- CTCCCTCCTGGCCATTCTGG − 3’
	5′- GGAAGCCGGAGTGCAGGTAT − 3’
ALP	5′- CTGGACCTCGTTGACACCTG − 3’
	5′- TCCGTCACGTTGTTCCTGTT − 3’
COL-1	5′- GAGGGCCAAGACGAAGACATC − 3’
	5′- CAGATCACGTCATCGCACAAC − 3’
OCN	5′- CTCACACTCCTCGCCCTAT −3’
	5′- TCTCTTCACTACCTCGCTGC −3’

### Statistical analysis

Each experiment was performed in triplicate and repeated at least three times. All values are presen-ted as mean ± standard deviation (Mean ± SD),and statistically performed data byGraphPad Prism 8 software. Comparison between two groups employed independent samples T-test, and One-way ANOVA and Tukey’s test was utilized to compare groups. *P* < 0.05 denoted that the difference is statistically significant.

## Results

### Construction of PDA-GO/CS composite  scaffold

1. PDA-GO synthesis

**Fig. 1:**
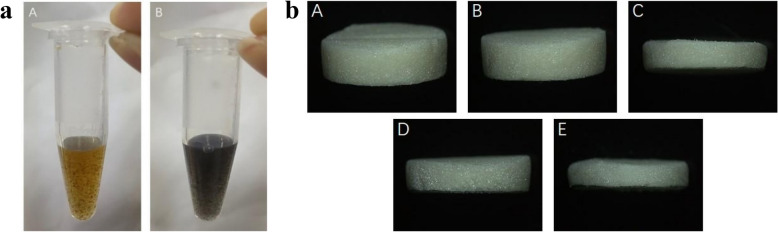
**a** Color change of GO and GO-PDA dispersion. *A*:GO;*B*:PDA-GO. **b** The general appearance of CS and different mass fraction PDA-GO/CS composite scaffolds. *A*:CS;*B*:0.1%PDA-GO/CS;*C*:0.3%PDA-GO/CS;*D*:0.5%PDA-GO/CS;*E*:0.7%PDA-GO/CS

As shown in Fig. 1(a), GO’s dispersion appears light brown. (B) PDA-GO dispersion appears black.

(2) PDA-GO/CS synthesis

As shown in Fig. 1(b), the support resembles a spongy porous structure that can be cut into different thicknesses as needed; The CS  scaffold is white, and the color of PDA-GO/CS composite scaffold deepens with the increase of PDA-GO quality.

### Detection of PDA-GO/CS composite scaffold

#### Fourier infrared detection results

**Fig. 2:**
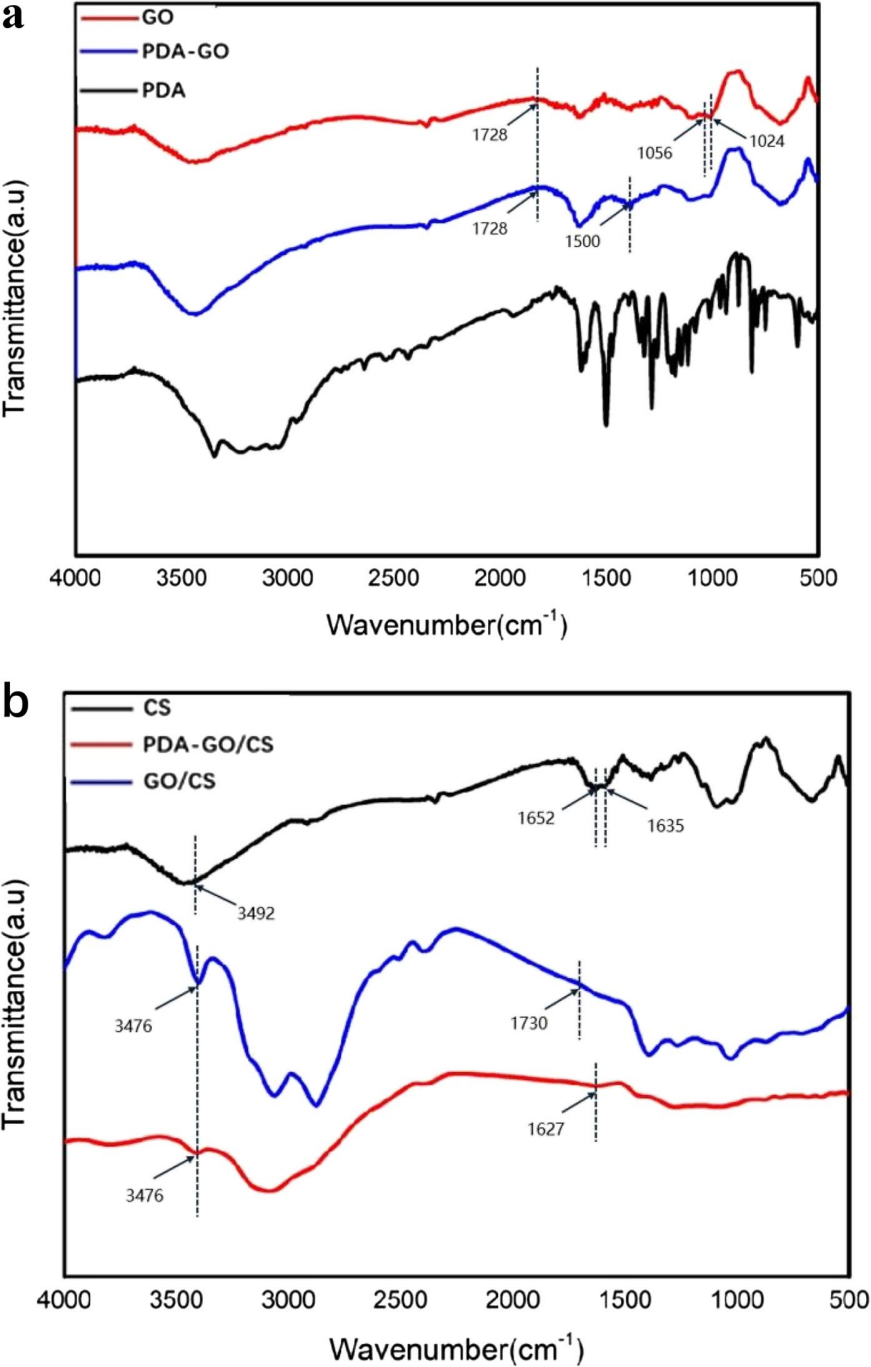
Fourier transform infrared spectroscopy analysis of GO, PDA-GO, PDA, CS, PDA-GO/CS, GO/CS

1. Infrared spectra of GO, PDA-GO and PDA, as shown in Fig. 2(a). For the GO sample, the stretching vibration at C=O (1728 cm^−1^), the stretching vibration of C-C from the unoxidized sp^2^C-C bond (1624 cm^−1^), and the stretching vibration of C-O (1056 cm^−1^ )[44]. For PDA-Go samples, the peak intensity of C=O at 1728 cm^−1^ was weakened, indicating that the oxygen-containing functional groups on GO surface were reduced to a certain extent after the reaction between PDA and GO. This is because GO is reduced by PDA [45]. At the same time, some new peaks appear in the spectrum of PDA-GO samples, such as the deformation vibration peak of N-H (1586 cm^−1^) and the stretching vibration peak of C=C (1500 cm^−1^). These results prove that GO reacts with PDA to generate PDA-GO [46].
2. Fourier infrared spectra of CS, GO/CS and PDA-GO/CS are shown in Fig. 2(b). For CS Sample, whose stretching vibration at C=O (1652 cm^−1^), bending vibration at -NH2 (1635 cm^−1^) [47]. For GO/CS samples, the characteristic peak of CS can be observed outside the stretching vibration peak of C=O (1730 cm^−1^) in GO. For PDA-GO/CS infrared spectra, the infrared absorption peaks of PDA-GO and the characteristic peaks of CS (about 1652 cm^−1^ and 1627 cm^−1^, respectively) can be observed. Compared with the PD-GO sample, the absorption bands near 3476 cm^−1^ become stronger and wider.

#### Antibacterial activity

1. Bacterial identification

**Fig. 3:**
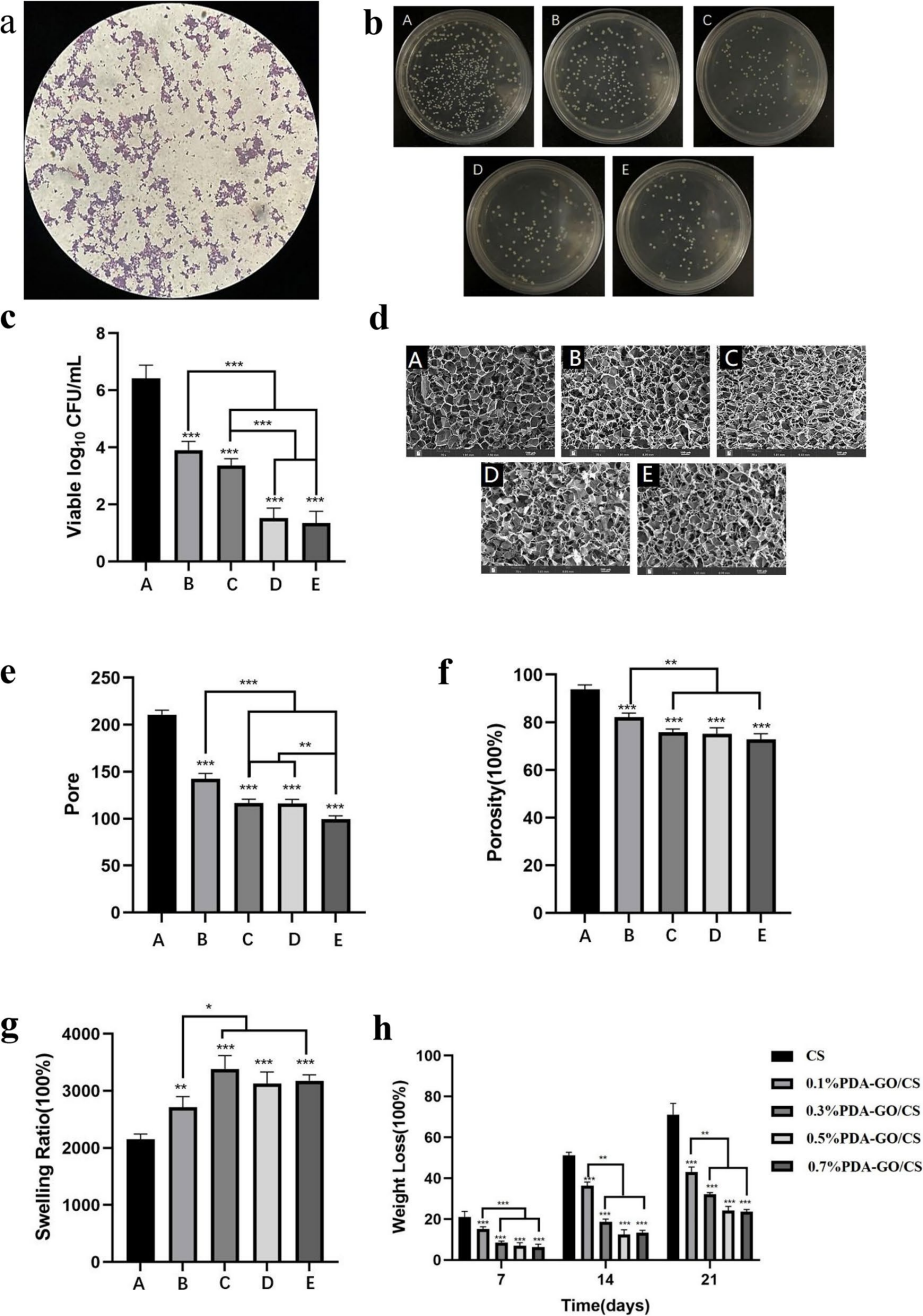
**a** Gram staining of *Sa*. **b** Qualitative data graph of antibacterial performance test of stents in each group. **c** Quantitative data graph of antibacterial performance test for each group of stents. **d** SEM images of the scaffolds in each group (scale bar: 100 μm) (**e**) Pore of scaffolds. **f** Porosities of scaffolds. **g** Swelling ratios of scaffolds. **h** Weight loss rate of scaffolds. (*p<0.05, **p<0.01, ***p<0.001)

As shown in Fig. 3(a), after the gram staining of Sa, the bacteria under the oil microscope showed purple staining, which was G^+^ bacteria, and the bacteria were spherical.

(2) Number of colonies on the scaffold surface

The CFU results of Sa of the five groups of stents are shown in Fig. 3(b). CS, 0.1%PDA-GO/CS, 0.3%PDA-GO/CS, 0.5%PDA-GO/CS and 0.7%PDA-GO/CS are represented by A, B, C, D and E, respectively. The number of colonies in group B, C, D and E was significantly less than that in group A.

The number of Sa colonies in the five groups of stents was shown in Fig. 3(c), and the experimental group was the same as above. Group A (6.41 ± 0.46)10^7^CFU/mL, Group B (3.9 ± 0.31)10^7^CFU/mL, group C (3.37 ± 0.24)10^7^CFU/mL, group D (1.52 ± 0.35)10^7^CFU/mL, group E (1.35 ± 0.41)10^7^CFU/mL. The number of colonies in group A and B, C, D and E were significantly different (*P* < 0.05), the colony number of group B was significantly different from group C, D and E (*P* < 0.05), there was statistical significance in the number of colonies in group C, D and E (*P* < 0.05), with the increase of PDA-GO mass fraction, the CFU value of PDA-GO/CS decreases.

#### SEM results

The SEM of the five groups of scaffolds is shown in Fig. 3(d), and the experimental grouping is the same as above. The five groups of scaffolds are porous mesh structures. The CS scaffolds were smooth, while PD-GO /CS scaffolds showed more folds. With the increase of PDA-GO content, the scaffold became more compact.

The aperture size of the five groups of scaffolds is shown in Fig. 3(e), and the experimental grouping is the same as above. The specific values of each group are shown in Table 2. The pore size difference between group A and group B, C, D and E was statistically significant (*P* < 0.05), the pore size difference between group B and group C, D and E was statistically significant (*P* < 0.05), the pore size difference between groups C, D and E was statistically significant (*P* < 0.05). With the increase of PDA-GO mass, the pore size of the stent gradually decreased, and the difference of the pore size of the C group and the D group was not statistically significant (*P* > 0.05).

**Table 2:**
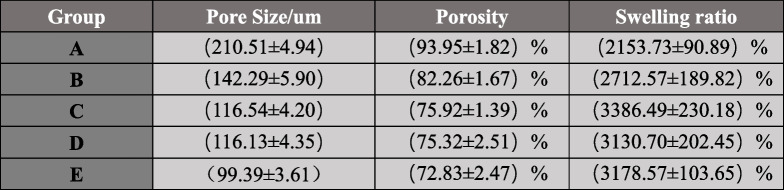
The pore size, porosity, and swelling ratio of each group of scaffolds.(Values are expressed as Mean ± SD)

#### Scaffold porosity

The porosity of the five groups of scaffolds is shown in Fig. 3(f), and the experimental groups are the same as above. The specific values of each group are shown in Table 2.The difference of porosity between group A and Group B, C, D and E was statistically significant (*P* < 0.05), the difference of porosity between group B and groups C, D and E was statistically significant (*P* < 0.05).

#### Swelling ratio of the scaffold

The swelling rates of the five groups of scaffolds are shown in Fig. 3(g), and the experimental groups are the same as above. The specific values of each group are shown in Table 2. The swelling ratios of group A and B, C, D and E were significantly different (*P* < 0.05), the swelling ratios of group B and groups C, D and E were significantly different (*P* < 0.05), group C had the highest swelling ratio.

#### Degradation of the scaffold

Degradation rates of the five groups of scaffolds were shown in Fig. 3(h), and the experimental groups were the same as above. The specific values of each group are shown in Table 3.The degradation rates of group A and B, C, D and E were significantly different (*P* < 0.05), the degradation rates of group B and groups C, D and E were significantly different (*P* < 0.05).

**Table 3:**
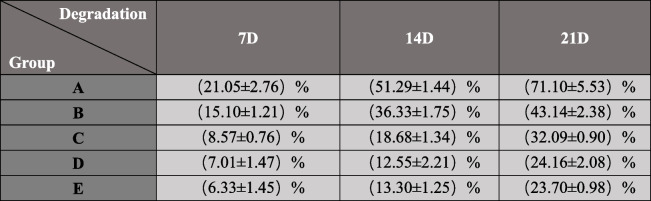
Degradation rate of each group of scaffolds. (Values are expressed as Mean ± SD)

### Isolation and culture of hDPSCs

**Fig. 4:**
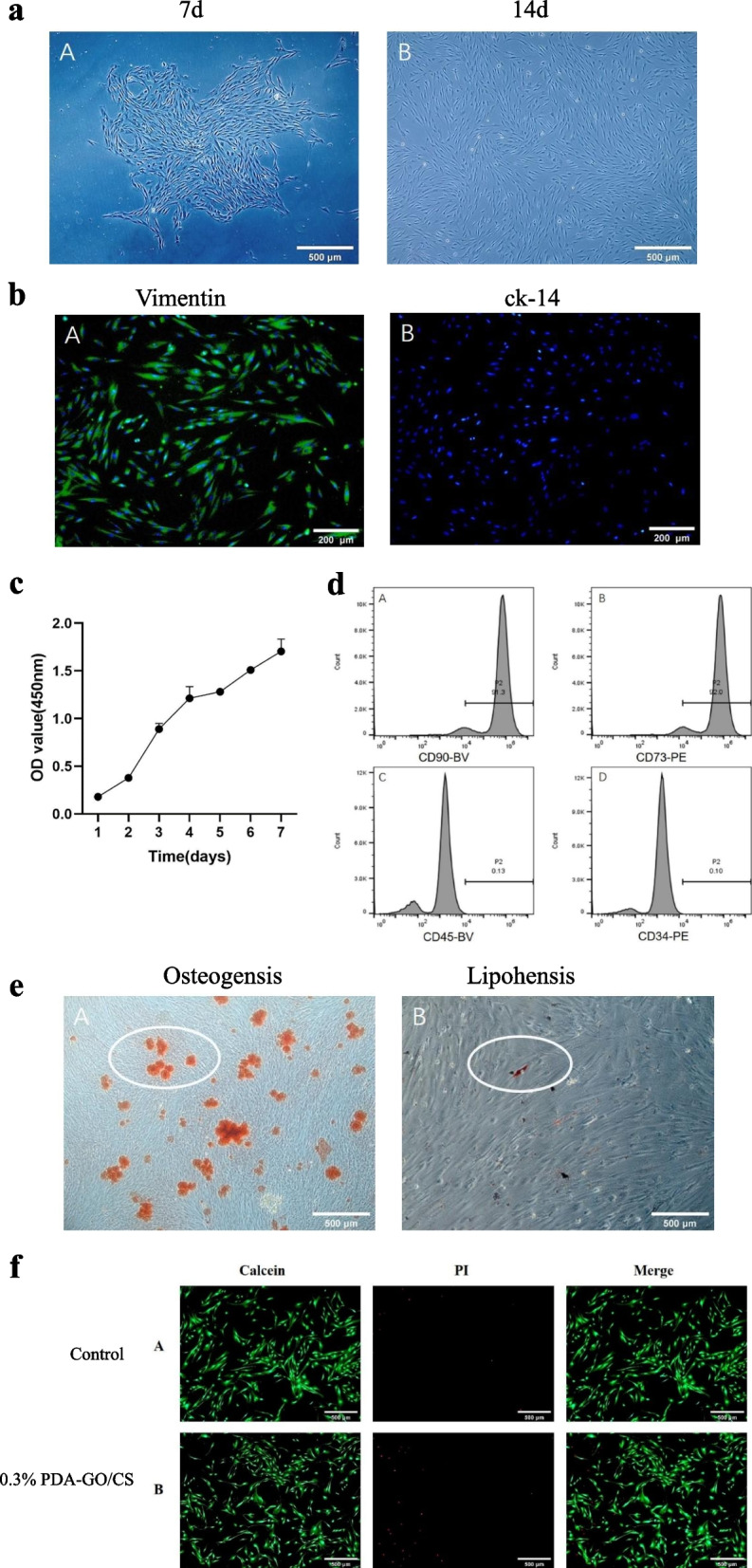
**a** Morphological observation of hDPSCs. (Scale bar: 500 μm) **b** Immunofluorescence staining of hDPSCs (Scale bar: 200 μm). **c** The 7-day growth curve of hDPSCs. **d** Flow cytometric analysis of the cell surface molecule expression of hDPSCs. **e** Staining of human dental pulp stem cells under different conditions for inducing differentiation (scale bar: 500 μm). **f** Live and dead staining results of cells on of Control group and 0.3%PDA-GO/CS group (Scale bar: 500 μm)

The primary hDPSCs were obtained by tissue block combined with enzyme digestion method. After 7 days of primary culture, the cells could be seen to crawl out of the tissue and grow adherent to the wall in a radial network arrangement centered on the tissue block. Most of the cells showed long spindle shape, while a few showed star shape, with slender cytoplasmic processes, clear outline and plump cytoplasm. After 14–20 days of culture, the primary hDPSCs were cultured and the cell density reached 70–80%. The cells showed spindle shape (Fig. 4(a)).

1. Immunofluorescence staining of antigen expression on cell surface

The immunofluorescence staining results of hDPSCs were shown in Fig. 4(b). Vimentin cells were stained green with positive expression. Nuclear DAPI staining was blue. Cell ck-14 was fluorescentially expressed in red, which was not shown in this figure, only blue in nuclear DAPI. These results indicated that the cultured cells were derived from mesenchyme.

(2) Cell growth curve drawing

The growth curve of hDPSCs was shown in Fig. 4(c). The cell growth curve was S-shaped, indicating that the cell growth was in good condition.

(3) Detection results of molecular markers by hDPSCs flow cytometry

The positive rate of hDPSCs surface antigen was determined by flow cytometry. The results showed that CD90 and CD73 showed antigen-positive expression rates of 91.3 and 92%, respectively. CD45 and CD34 showed antigen-negative expression rates of 0.13 and 0.10% (Fig. 4(d)), indicating that the cells obtained from primary culture were hDPSCs.

(4) Osteogenic induction differentiation

Alizarin red dye was used to stain the fixed hDPSCs, and after three steaming, a large area of red mass could be seen at 50 times of microscope, which was the formation area of calcified nodules, indicating that the hDPSC used in this experiment had good osteogenic ability (Fig. 4(e)).

(5) Lipid induction differentiation

The fixed hDPSCs were stained with oil red O dye, and the bright red coloring points were visible at 50 times of the mirror, namely lipid droplets, indicating that the hDPSCs cultured in this experiment had lipid formation ability (Fig. 4(e)).

### In vitro cytotoxic morphology will

The images were taken under a fluorescent inverted microscope, as shown in Fig. 4(f). The Control group and 0.3%PDA-GO/CS group were represented by A and B, respectively. Fluorescence expression in living cells was green (Calcein) and red (PI) in dead cells. The results showed that the living cells in groups Control and 0.3%PDA-GO/CS showed long spindles, while the number of dead cells was low.

### Cytoskeleton

**Fig. 5:**
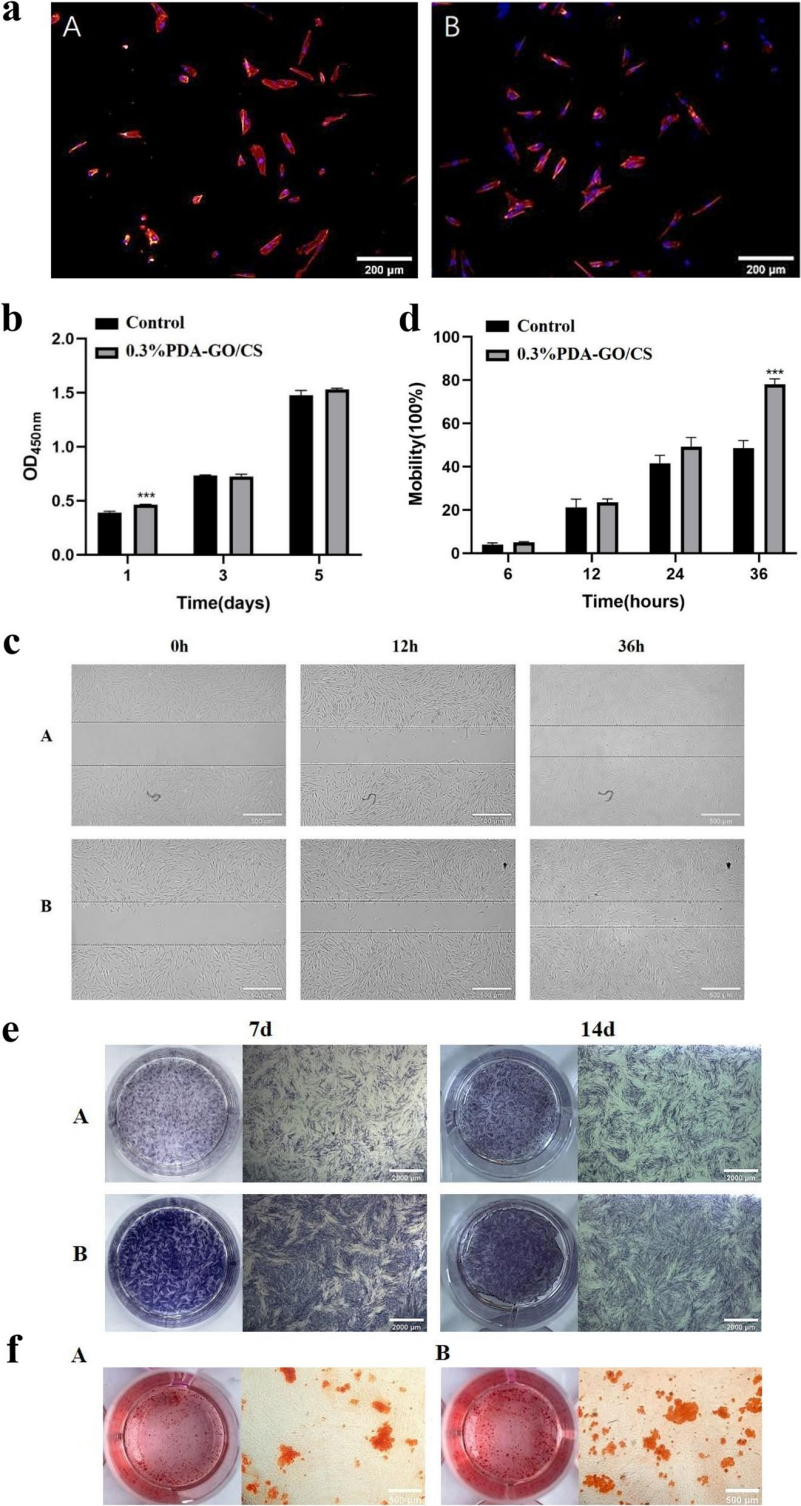
**a** The cytoskeleton staining results of A group and B group (Scale bar: 200 μm) **b** Results of proliferative ability in A group and B group. **c** Qualitative results of migration ability in A group and B group (Scale bar: 500 μm). **d** Quantitative results of migration ability in A group and B group. **e** Alkaline phosphatase staining results of in A group and B group (Scale bar: 2000 μm) **f** Alizarin red staining results of in A group and B group (Scale bar: 500 μm) (*p<0.05, **p<0.01, ***p<0.001)

Sample extracts were used for cell culture. After the cells were inoculated into the 96-well plate, the sample extract was changed after the cells adhered to the wall, and the culture was continued. After 72 h, the skeleton of the cells was observed under the fluorescence microscope, as shown in Fig. 5(a). The Control(A) group and 0.3%PDA-GO/CS(B) group were represented by A and B, respectively. The cytoskeleton is red and the nucleus is blue. hDPSCs showed typical long spindle shape, cytoskeleton could be observed, and the round nucleus was located in the center of the cell. The whole cell was well extended, and the cell morphology of group B was not significantly different from that of group A.

### Effects of PDA-GO/CS on proliferation capacity of hDPSCs

The hDPSCs were cultured in 0.3%PDA-GO/CS extract, and the proliferation capacity of the cells was detected by CCK-8 method. OD values were detected at the 1st, 3rd and 5th days, respectively. As shown in Fig. 5(b), OD value in the 1st day: 0.3% PDA-GO/CS group was higher than that in the Control group, and the difference between the two groups was statistically significant (*P* < 0.05). At day 3 and 5, the difference between the two groups was not statistically significant (*P* < 0.05).

### Effects of PDA-GO/CS on the migration ability of hDPSCs

The effect of scaffold on cell migration was detected by scratch test. At 0, 6, 12, 24, 36 and 48 hours after the scratch, the scratched areas of different groups were photographed under the same field of vision at different times, as shown in Fig. 5(c). The Control group and 0.3%PDA-GO/CS group were represented by A and B respectively, and cell migration images at 0, 12 and 36 h were listed. It can be seen that at 36 h, the scratch area of all groups decreased compared with 0 h, while the scratch area of B decreased more. Image J software was used to calculate the scratch area of group A and Group B in different time periods, and the mobility of each group at each time point was calculated according to formula (4), and statistical analysis was made, as shown in Fig. 5(d). It can be seen from the change of the curve over time that B has the highest mobility and has A difference with A at 36 h (*P* < 0.05).

### Effects of PDA-GO/CS on osteogenic differentiation of hDPSCs

1. Alkaline phosphatase staining

The hDPSCs were cultured with 0.3%PDA-GO/CS extract, and osteogenic differentiation was induced. The Control group and 0.3%PDA-GO/CS group were represented by A and B, respectively, as shown in Fig. 5(e). After 7 and 14 days of continuous induction, alkaline phosphatase staining results showed that group A and group B showed positive expression, and the stained area showed a vortex under the microscope. Alkaline phosphatase staining on the 7th and 14th day in group B was darker than that in group A.

(2) Alizarin red staining

Alizarin red staining was performed 21 days after mineralization induction. The Control group and 0.3%PDA-GO/CS group were represented by A and B, as shown in Fig. 5(f). Dark red dotted deposits of different sizes were visible to the naked eye. Microscopic observation showed that both groups had red mineralized nodules with deep staining in the center of nodules. Compared with group A, group B had more and more dispersed mineralized nodules.

(3) Expression of osteogenic genes

**Fig. 6:**
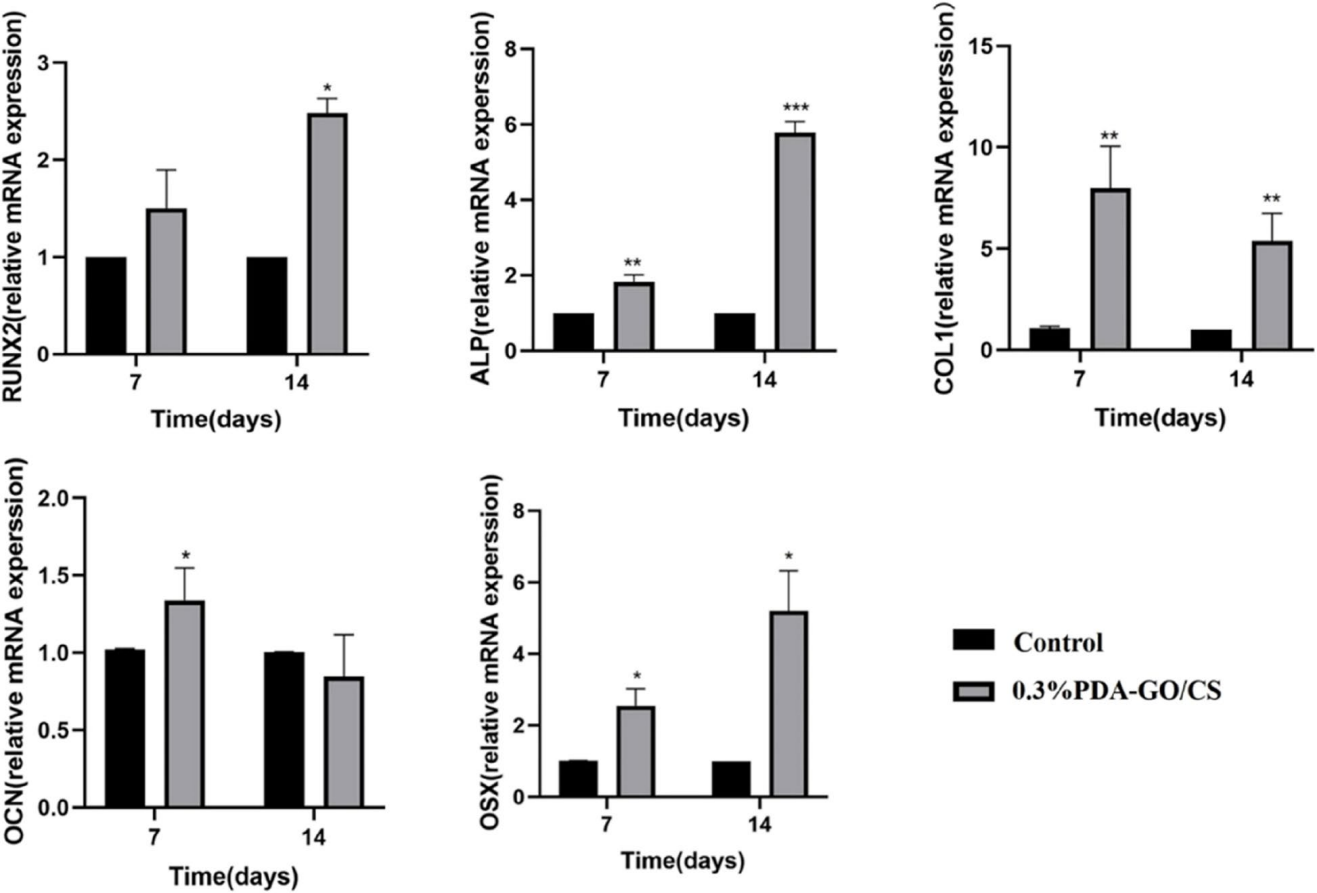
Expression of osteogenic differentiation genes in Control group and 0.3%PDA-GO/CS group (*P < 0.05)

The cells in Control group and 0.3%PDA-GO/CS group were cultured for 7 and 14 days, respectively. qRT-PCR was used to detect the changes of gene expression levels related to osteogenic differentiation. The results showed that different genes showed different trends in the 7th and 14d numbers of culture. Compared with the Control group, the relative expression of osteogenic genes in 0.3%PDA-GO/CS was shown in Fig. 6. RUNX2 gene expression was up-regulated: on the 14th day, 0.3%PDA-GO/CS was significantly different from Control group (*P* < 0.05). ALP gene expression was up-regulated: on the 7th day, 0.3%PDA-GO/CS was significantly different from Control group (*P* < 0.05), and on the 14th day, 0.3%PDA-GO/CS was significantly different from Control group (*P* < 0.05). COL1 gene expression was up-regulated: at the 7th and 14th day, 0.3%PDA-GO/CS was significantly different from Control group (*P* < 0.05). OCN gene expression was up-regulated: on the 7th day, 0.3%PDA-GO/CS was significantly different from Control group (*P* < 0.05). OSX gene expression was up-regulated: on the 7th and 14th day, 0.3%PDA-GO/CS was significantly different from the Control group (*P* < 0.05).

## Discussion

rGO can be generated by thermal reduction, electric reduction or chemical reduction, among which chemical reduction is the most commonly used method. PDA is a kind of natural material with excellent performance [43], good hydrophilicity, adhesiveness, non-toxicity, degradability and biocompatibility. In this experiment, PDA is selected to GO reduction. The GO dispersion appears light brown and the PDA-GO dispersion appears black. The successful preparation of PDA-GO can be confirmed by Fourier infrared detection. Through FTIR observation of CS, GO/CS and PDA-GO/CS, the corresponding group changes were observed, which could confirm the synthesis of PDA-GO/CS composite scaffolds. By SEM observation of the scaffold, it can be seen that PDA-GO/CS composite scaffold has many folds relative to CS, which can also confirm the synthesis of PDA-GO/CS composite scaffold.

The optimal mass fraction of PDA-GO/CS composite scaffolds was sought, and the literature and pre-experimental results were consulted. In this experiment, the mass fraction was set to be 0.1, 0.3, 0.5 and 0.7% for PDA-GO to be combined with CS scaffolds. The main adsorption mechanism is π-π interaction due to partial elimination of oxygen-containing functional groups on rGO surface. Hydrophobic interactions are involved in the antibacterial activity of this 2D nanomaterial and promote the destruction of bacterial membranes [48]. This may be due to the increase of PDA-GO, which makes PDA-GO/CS more effective contact with bacteria, so as to obtain better antibacterial effect.

The SEM results showed that the PDA-GO/CS composite scaffolds showed fold shape. These folds can increase the specific surface area of scaffolds. Kai Zhou et al. [49] found that oxygen-containing groups on the surface of rGO provide binding sites for more cells, which is conducive to cell adhesion. Irene Buj-Corral et al. [50] found that high surface roughness of scaffolds was conducive to bone integration. Pore size is essential for the formation of bone tissue, and a pore size more than100μm is conducive to the migration and proliferation of osteoblasts and mesenchymal cells [51]. The suitable porosity of scaffolds in bone tissue engineering is 30–90 % [52]. SEM results of 0.3%PDA-GO/CS scaffold showed that the addition of PDA-GO increased the surface roughness of CS scaffold, which was conducive to scaffold implantation. The pore size and porosity of 0.3%PDA-GO/CS are (116.54 ± 4.20) μm and (75.92 ± 1.39) %, which are consistent with bone tissue engineering. The scaffold with high swelling ratio was beneficial to cell adhesion and proliferation, and the swelling rate (3386.49 ± 230.18) % of 0.3%PDA-GO/CS group was higher than that of CS group, and the difference was statistically significant (P<0.05). The degradation rate of 0.3%PDA-GO/CS group was significantly lower than CS group, and the difference was statistically significant (P<0.05). To sum up, 0.3%PDA-GO/CS group was selected for the following experiment.

hDPSCs are a promising multifunctional source for bone regeneration with high osteogenic potential. They can differentiate into osteoblasts, promote vascularization, and modulate immune functions, which are crucial for fostering new bone formation. hDPSCs also possess unique capabilities in regenerating complex tissues such as the dentin-pulp complex, making them a promising source for intricate tissue regeneration. In comparison to BMSCs, hDPSCs offer advantages like ease of extraction and preservation from dental tissues, low immunogenicity, and reduced ethical concerns [53]. In this study, hDPSCs were used as seed cells to evaluate the effects of 0.3%PDA-GO/CS composite scaffolds on proliferation, migration and osteogenic differentiation. Compared with the Control group, 0.3%PDA-GO/CS group promoted cell proliferation at the 1st day, and there was no statistical difference between the two groups at the 3rd and 5th day, indicating that the composite scaffold did not inhibit cell proliferation. Alkaline phosphatase staining and alizarin red staining were performed to study the mineralization potential of the two groups of cells. At two different time periods, alkaline phosphatase staining was deeper in the composite scaffold group than in the Control group. Alizarin red staining in the composite scaffold group was darker than that in the Control group. These results indicated that composite scaffolds could promote osteogenic differentiation of cells.

The expression levels of osteogenic genes RUNX2, ALP, OSX, COL-1 and OCN were detected by RT-qPCR. ALP is an indicator of early osteogenic differentiation of cells, and the higher the activity of ALP, the better the ability of osteogenic differentiation of cells [54]. On the 7th and 14th days of osteogenesis induction, the content of ALP in cells in the composite scaffold group was significantly higher than that in the Control group. Indicators of late osteoblastic differentiation of OCN cells [55]. On the 7th day of osteogenic induction, OCN content in the composite scaffold group was significantly higher than that in the Control group. RUNX2 is a specific transcription factor that plays a regulatory role in osteoblasts and facilitates osteogenic differentiation of cells [56]. On day 7 of osteogenic induction, the content of RUNX2 in cells of the composite scaffold group was significantly higher than that of the Control group. COL1 is an extracellular matrix protein that promotes osteoblast adhesion and differentiation [57]. On the 7th and 14th days of osteogenic induction, the content of COL1 in cells in the composite scaffold group was significantly higher than that in the Control group. The study [58] indicates that Osterix (Osx) is a crucial mesenchymal transcription factor involved in the processes of osteogenesis and odontogenesis. On the 7th and 14th days of osteogenesis induction, OSX content in 0.3%PDA-GO/CS group was significantly higher than that in Control group. The difference was statistically significant (*P* < 0.05).

0.3%PDA-GO/CS composite scaffolds promoted the differentiation of hDPSCs into osteoblasts in different aspects. Three-dimensional CS scaffold material, similar to natural extracellular matrix [59], is conducive to cell adhesion and proliferation. GO is a new nanomaterial that has been extensively studied in recent years, and it has been proved that this material can promote osteogenic differentiation and in vivo osteogenesis [48]. Compared with CS, 0.3%PDA-GO/CS composite scaffold has a higher folding rate, which is conducive to cell adhesion growth. Meanwhile, the composite scaffold can promote the differentiation of hDPSCs into osteoblasts. At the same time, the shape of 0.3%PDA-GO/CS composite scaffold has a high degree of plasticity, and the corresponding shape can be shaped according to the needs. This feature makes it more convenient for clinical application. The 0.3%PDA-GO/CS composite scaffold could better promote the osteogenic differentiation of hDPSCs.

This study investigated the successful fabrication of the PDA-GO/CS composite scaffold. We assessed the pore size, porosity, swelling ratio, and degradation rate of PDA-GO/CS scaffolds with different mass ratios, alongside evaluating their antibacterial properties. Our findings revealed that the 0.3% PDA-GO/CS composite scaffold enhanced the antibacterial activity and hydrophilicity of CS while reducing the degradation rate. In vitro studies with hDPSCs demonstrated that the PDA-GO/CS composite scaffold promoted cell adhesion, proliferation, migration, and mineralization. The research primarily focused on the physicochemical properties and biocompatibility of the PDA-GO/CS composite scaffold. However, further testing for antibacterial efficacy against various bacteria and establishing animal models to support our results through in vivo studies are warranted. The PDA-GO/CS composite scaffold exhibits outstanding biocompatibility and bioactivity, offering promising outcomes in bone tissue engineering.

## Conclusions

In summary, the PDA-GO/CS composite scaffold exhibits favorable physicochemical properties and biocompatibility, promoting adhesion, proliferation, migration, and differentiation of hDPSCs. It stands as a novel scaffold material suitable for cell culture, holding promising prospects in bone defect repair and the advancement of bone tissue regeneration in the field of tissue engineering.

## Data Availability

All data generated or analysed during this study are included in this published article.

## Abbreviations

α-MEM: α-Modified Eagle’s Medium
ALP: alkaline phosphatase
cDNA: complementary DNA
COL1: collagen type 1
CS: chitosan
DAPI: 4, 6-diamidino-2-phenylindole dihydrochloride
ddH_2_O: double distillated water
DEPC: diethylprocarnate
hDPSCs: human dental pulp stem cells
FBS: fetal bovine serum
GAPDH: glyceraldehyde phosphate dehydrogenase
GO: graphene oxide
OCN: osteocalcin
DMP1: dentin Matrix Protein 1
OD: optical density
PBS: phosphate-buffered saline
PDA: polydopamine
qRT-PCR: real time flourescent quantitative polymerase chain reaction
RNA: ribonucleic acid
RNase: ribonuclease
rpm: round per minute
RT: reverse transcription
RUNX2: runt-related transciption factor 2
FTIR: fourier transform infrared spectrometer
